# Use of Bacterial Toxin–Antitoxin Systems as Biotechnological Tools in Plants

**DOI:** 10.3390/ijms251910449

**Published:** 2024-09-27

**Authors:** Bernardo Rodamilans, Xiaofei Cheng, Carmen Simón-Mateo, Juan Antonio García

**Affiliations:** 1 Centro Nacional de Biotecnología (CNB-CSIC), Campus Universidad Autónoma de Madrid, Darwin 3, 28049 Madrid, Spain; csimon@csic.es (C.S.-M.); jagarcia@cnb.csic.es (J.A.G.); 2 College of Plant Protection, Northeast Agricultural University, Harbin 150030, China; xfcheng@neau.edu.cn

**Keywords:** toxin, antitoxin, protease, NIa, potyvirus, antiviral

## Abstract

Toxin–antitoxin (TA) systems in bacteria are key regulators of the cell cycle and can activate a death response under stress conditions. Like other bacterial elements, TA modules have been widely exploited for biotechnological purposes in diverse applications, such as molecular cloning and anti-cancer therapies. However, their use in plants has been limited, leaving room for the development of new approaches. In this study, we examined two TA systems previously tested in plants, MazEF and YefM-YoeB, and identified interesting differences between them, likely related to their modes of action. We engineered modifications to these specific modules to transform them into molecular switches that can be activated by a protease, inducing necrosis in the plant cells where they are expressed. Finally, we demonstrated the antiviral potential of the modified TA modules by using, as a proof-of-concept, the potyvirus plum pox virus as an activator of the death phenotype.

## 1. Introduction

Bacteria and archaea encode hundreds of toxin–antitoxin (TA) intracellular modules involved in various cellular processes, which become particularly relevant under stress conditions. TA systems are classified into eight groups (types I–VIII) based on the chemical nature of the antitoxins and their mode of action [1,2]. Type II TA systems, the first to be characterized and the most common [3], are organized as operons with the antitoxin gene preceding the toxin gene. The direct protein–protein interaction between the two components forms a harmless complex [4]. TA systems, especially type II, have garnered significant interest in recent decades due to their ability to kill cells where they are expressed, leading to their exploitation in various biotechnological applications such as DNA cloning [5], phage resistance [6,7], drug development [8], anticancer therapies [9], or antiviral tools [10,11]. However, the application of bacterial TA systems in plants has been relatively modest.

One of the earlier uses of bacterial toxins in plant biotechnology involved Barnase/Barstar from *Bacillus amyloliquefaciens*. Although not a true TA system since the proteins are not expressed as an operon, its control allowed the generation of male-sterile plants and restoration phenotypes, which were useful for engineering genetically modified plants [12,13]. Cell ablation and male sterility have also been achieved through the expression of the MazF toxin from *Escherichia coli* [14]. MazF is part of the MazEF TA system, one of the first described type II TA bacterial modules, known for its broad involvement in stress responses [15,16]. MazF exerts its activity by cleaving mRNA at ACA sequences in a ribosome-independent manner, leading to the inhibition of protein synthesis [17]. Another TA module reported to be useful in plants is the YefM-YoeB complex from *Streptococcus pneumoniae*. In *Arabidopsis thaliana*, a YoeB-GFP fusion was shown to induce a death phenotype that could be counteracted by the expression of the cognate YefM antitoxin [18,19]. YefM-YoeB is also a type II TA system, but unlike MazEF, it functions in association with ribosomal subunits to block translation initiation [20].

In this work, we explored the potential of using TA systems in a broader biotechnological context by coupling them with a protease activation switch to expand their capabilities. We tested two TA systems used in plants, MazEF and YoeB-YefM, and found that they functioned quite differently from one another in *Nicotiana benthamiana*. Additionally, we observed that the YoeB-YefM system behaved differently from what was described in *A. thaliana*. We engineered specific constructs for each system and successfully developed molecular OFF/ON systems based on the Nuclear Inclusion A endopeptidase (NIapro) from potyviruses. Furthermore, we evaluated the potential of these modified bacterial toxins as antiviral tools using the potyvirus plum pox virus (PPV) as a proof-of-concept.

## 2. Results

### 2.1. Testing TA Systems in Nicotiana benthamiana

**Figure 1:**
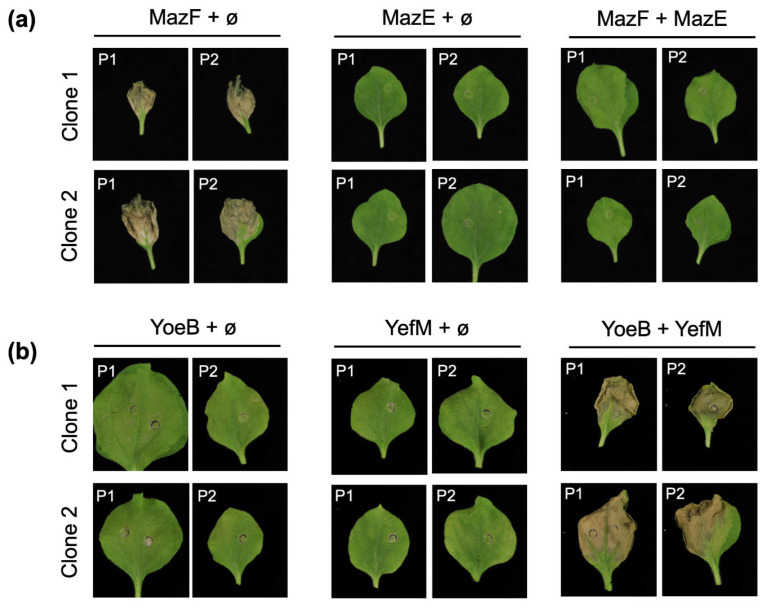
Testing two different TA systems in *Nicotiana benthamiana* plants. Photos from plants expressing the MazEF (**a**) and YefM-YoeB (**b**) modules were taken at 10dpa, and two representative images in each case are shown. The plant number is marked on the top left corner. Plasmids were expressed as indicated, and empty plasmid pGWB402Ω (ø) was included to compensate for agrobacterium amounts. All mixes were prepared including P19 as RNA silencing suppressor.

To explore the use of TA modules in plants for novel biotechnological applications, we selected two pairs of TA systems: MazEF from *E. coli* and YefM-YoeB from *S. pneumoniae*. These systems had previously been tested in *Nicotiana tabacum* and *A. thaliana*, respectively [14,19]. We synthesized the corresponding toxin genes for each system, introducing an intron in the middle of their sequences to prevent cloning issues in bacteria [21], and we also synthesized the cognate antitoxin genes. Plasmids were transformed into agrobacteria for the transient expression of the corresponding proteins. All plasmids were co-expressed with the RNA silencing suppressor P19 to maximize protein expression over time. We agroinfiltrated eight plants with the indicated mixtures (Figure 1), four plants per clone, and monitored them over time, up to 10 days post-agroinfiltration (dpa).

**Figure 2:**
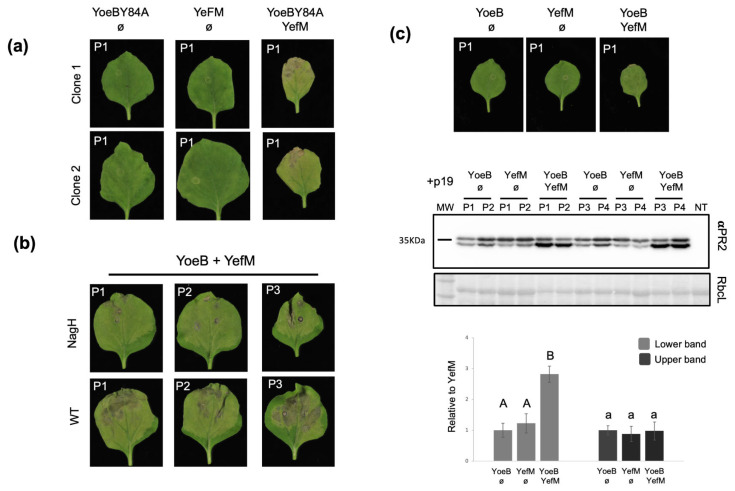
Characterizing the necrotic phenotype of the YefM-YoeB system. (**a**) Effect of the inactive YoeB toxin (YoeBY84A) in the YefM-YoeB module. Photos were taken at 10dpa, and one representative image from each clone was selected. The plant number is marked on the top left corner. Mixes were prepared as indicated, and empty plasmid pGWB402Ω (ø) was included to compensate for agrobacterium amounts. All mixes were prepared including P19 as RNA silencing suppressor. (**b**) Expression of the YefM-YoeB system in NahG and wild-type *N. benthamiana* plants. Photos from the plants agroinfiltrated with YefM and YoeB were taken at 10dpa. Three representative images from eight plants are shown. All mixes were prepared including P19 as RNA silencing suppressor. The plant number is marked on the top left corner. (**c**) Photos were taken at 5dpa, and one representative plant is shown (upper panel). Wild-type plants were agroinfiltrated with the indicated mixes, and empty plasmid pGWB402Ω (ø) was included to compensate for agrobacterium amounts. In the middle panel, Anti-PR2 immunoblot analysis of protein extracts from tissue collected from (**a**); NT, nontreated plant; MW, molecular weight; a Ponceau red-stained blot (RbcL) showing the small subunit of the Rubisco is displayed below the membrane as a loading control. A densitometry analysis of the bands relative to the YefM samples is shown in the lower panel; lowercase or uppercase letters indicate significant differences or not based on one-way ANOVA for the upper or lower band, respectively.

As anticipated, the expression of the MazF toxin induced a death phenotype, which was counteracted by the expression of the cognate MazE antitoxin. Additionally, the MazE antitoxin alone did not produce any necrotic effect (Figure 1a). This result was consistent with findings previously described in *N. tabacum* [14]. Surprisingly, for the YefM-YoeB system, the expression of the YoeB toxin, known to kill *A. thaliana* plants [16], or the YefM antitoxin did not produce any distinguishable phenotype. However, simultaneous expression of both proteins, which was harmless in *A. thaliana* [19], induced a death phenotype in the agroinfiltrated leaves similar to that observed in MazF-treated plants (Figure 1b). This result suggests that the toxin alone is not responsible for the death phenotype. Supporting this hypothesis, an experiment with an inactive mutant of the bacterial toxin yielded similar results. In this case, we synthesized an inactive YoeB toxin with a mutation in amino acid 84 (YoeBY84A) as described [22] and performed a comparable transient expression experiment in *N. benthamiana* plants. As before, expression of the mutant toxin or the antitoxin did not produce any notable phenotype in the agroinfiltrated area (Figure 2a).

However, the expression of the inactive mutant and the antitoxin did cause necrosis in the leaves at 10dpa, similar to what was observed when the wild-type toxin was co-expressed with the antitoxin. This result strongly supports the idea that the action of the toxin itself is not crucial for triggering the necrotic phenotype and suggests that the plant recognizes the TA complex and mounts an effector-triggered immunity (ETI)-like response that leads to cellular death. If this is the case, it is likely that salicylic acid (SA) is involved in the process, as reported in [23]. To verify this, we performed transient expression experiments with YefM-YoeB and MazEF modules in *N. benthamiana* wild-type plants and in transgenic plants (NahG), which express the bacterial salicylate hydroxylase gene nahG [24] and produce low levels of SA. Our results showed that, for the YefM-YoeB module, NahG plants exhibited milder necrosis compared to wild-type plants (Figure 2b). The necrosis caused by MazF was similar in both wild-type and NahG plants (Figure S1 in File S1 in the Supplementary Materials). Additionally, we analyzed the levels of pathogenesis-related protein 2 (PR2) in *N. benthamiana* agroinfiltrated plants. PR2 is a protein induced upon SA upregulation [25]. We performed agroinfiltration with the indicated mixtures (Figure 2c) and, at 5dpa, before necrosis was apparent in the leaves, we collected tissue and analyzed PR2 expression by Western blot. Densitometry analysis revealed a significant increase in PR2 levels in plants agroinfiltrated with both the toxin and antitoxin together compared to plants agroinfiltrated with either one alone. These results support the notion that SA induction is involved in the necrotic response to the co-expression of the YefM-YoeB module.

### 2.2. Exploring the Use of the MazEF System as a Biotechnological Tool

To further study the use of TA systems as functional elements in plant biotechnology, we aimed to transform them into molecular switches controlled by a protease. For this purpose, we selected NIapro, a well-characterized 3C-like endopeptidase [26] encoded by viruses of the *Potyviridae*, which is significant due to its social and economic impact on agriculture [27]. Upon activation of the toxin by the protease, a clear phenotypic response is expected, making it possible to use this system as a genetic circuit with a simple YES OR NO logic for synthetic biology applications [28].

**Figure 3:**
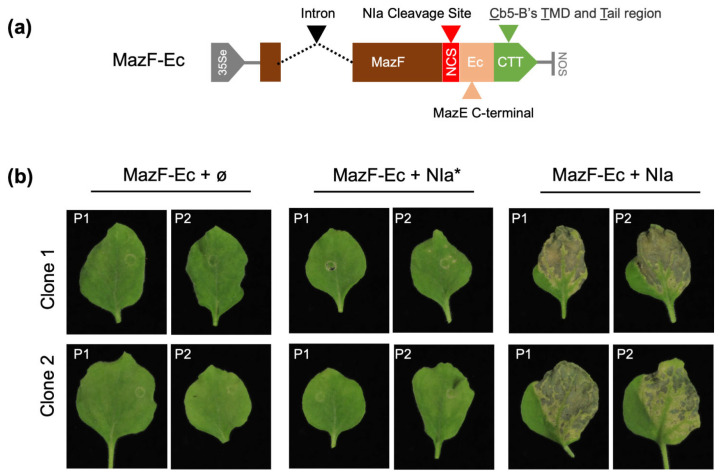
Adapting MazEF for biotechnological applications. (**a**) Schematic representation of the construct engineered based on the MazEF module, MazF-Ec; 35Se, enhanced 35S promoter; NOS, nopaline synthase terminator. (**b**) Expression of the MazF-Ec construct with wild-type (NIa) or mutated (NIa*) PPV NIa protease. Photos were taken at 10dpa, and two representative images from each clone were selected. The plant number is marked on the top left corner. Mixes were prepared as indicated using pGWB402Ω as empty plasmid (ø) and including P19 as RNA silencing suppressor.

For the MazEF module, we adopted the strategy used for the human Hepatitis C virus [10]. The MazF toxin was expressed as a fusion with the C-terminal part of the MazE antitoxin (Ec), which is sufficient for toxin binding and inactivation. A cleavage site for the viral endopeptidase NIapro from the potyvirus PPV (NCS) was inserted between the MazF toxin and the antitoxin. To ensure that MazF remains active and induces a death phenotype upon release of Ec, an anchor peptide (CTT) was included to keep the cleaved antitoxin region attached to the endoplasmic reticulum [29] (MazF-Ec; Figure 3a).

As in previous experiments, the new module was tested in *N. benthamiana* plants using transient expression. For system activation, the MazF-Ec construct was co-agroinfiltrated with NIa, a Viral Protein genome-linked (VPg)-NIapro product naturally produced during viral infections. Co-agroinfiltration of MazF-Ec with either an empty vector or an inactive NIa mutant protein (NIa*) [30] served as negative controls. P19 was included in each mixture to ensure high protein expression levels. Eight plants were agroinfiltrated, with four plants per clone, and were monitored over time. Leaves co-agroinfiltrated with an empty vector or the NIa mutant showed no specific damage after 10 days. In contrast, plants co-agroinfiltrated with wild-type NIa exhibited signs of necrosis at 5dpa and a clear death phenotype by 10dpa (Figure 3b). These results indicate that the system was adequately expressed in a switch-off position and was successfully activated upon expression of the viral endopeptidase.

### 2.3. Designing a Different Approach to Use the YefM-YoeB Module

**Figure 4:**
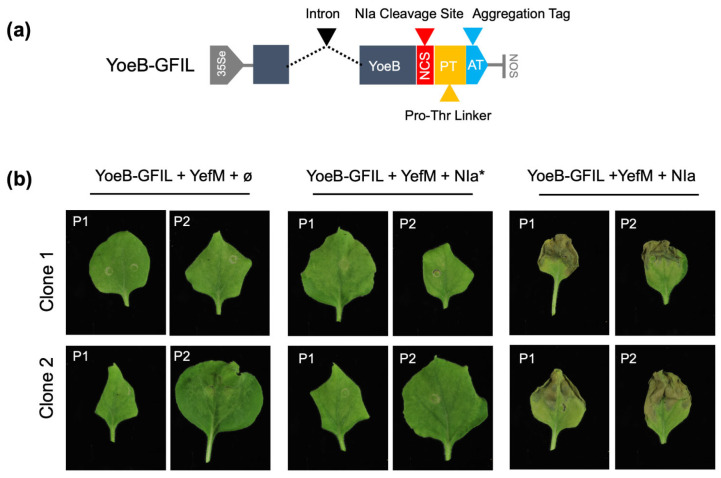
Adapting YefM-YoeB for biotechnological applications. (**a**) Schematic representation of the construct engineered based on the YefM-YoeB module, YoeB-GFIL; 35Se, enhanced 35S promoter; NOS, nopaline synthase terminator. (**b**) Expression of the YoeB-GFIL and YefM constructs with wild-type (NIa) or mutated (NIa*) PPV NIa protease. Photos were taken at 10dpa, and two representative images from each clone were selected. The plant number is marked on the top left corner. Mixes were prepared as indicated using pGWB402Ω as empty plasmid (ø) and including P19 as RNA silencing suppressor.

The results obtained with the YoeB toxin led us to seek a different method to design a molecular switch that could be activated by NIapro. In this case, the off position of the system could not be achieved through the expression of the antitoxin, as its expression is necessary for the necrotic response in the plant. Based on the hypothesis that the necrotic phenotype is triggered by the recognition of the complete TA protein complex, we engineered a method to keep both proteins physically separated by incorporating a C-terminal aggregation tag (AT) into the YoeB toxin. This tag consists of amino acids Gly-Phe-Ile-Leu (GFIL) expressed twice in tandem. Although this tag was described for bacteria [31], it had not been tested in plants. To facilitate aggregation, we included a rigid Pro/Thr linker before the tag (PT). A PPV-NIa cleavage site was inserted between YoeB and the PT linker (NCS) (YoeB-GFIL; Figure 4a).

As with the MazEF system, the YoeB-GFIL construct was tested through transient expression in *N. benthamiana* plants, co-agroinfiltrated with either an empty vector, an NIa inactive mutant (NIa*), or a wild-type NIa protein. YefM and P19 were included in all cases to enable the necrotic response and to suppress RNA silencing, respectively. Eight plants were agroinfiltrated, with four plants per clone, and the phenotype was monitored over time. Plants co-agroinfiltrated with an empty vector or the mutant NIa (NIa*) did not exhibit any specific phenotype by 10dpa. In contrast, plants co-agroinfiltrated with the plasmid encoding wild-type NIa showed incipient necrosis at 6dpa, which became apparent by 10dpa (Figure 4b). These results indicate that the YefM-YoeB module can also function as a molecular switch in *N. benthamiana* and highlight the potential of using aggregation tags in plants for the expression of harmful heterologous proteins.

### 2.4. Testing the Potential of TA Systems as Antiviral Tools

**Figure 5:**
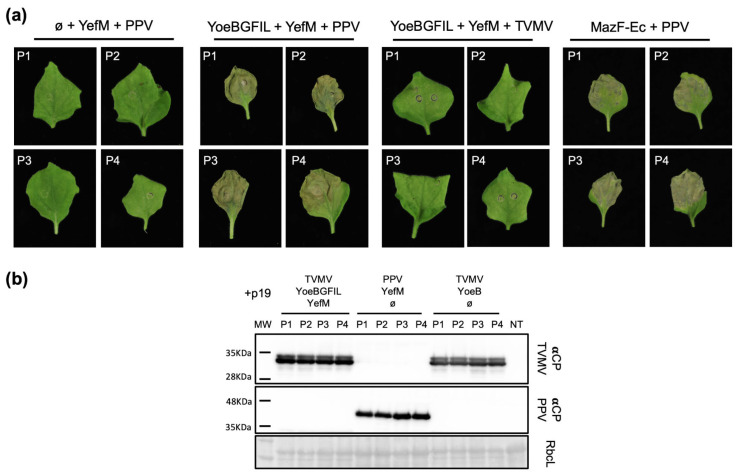
Virus sentinel capacities of the TA modules. (**a**) *Nicotiana benthamiana* plants were agroinfiltrated with the indicated mixes using pGWB402Ω as empty plasmid (ø) and including P19 as RNA silencing suppressor. Photos were taken at 10dpa, and four representative images from each mix were selected. The plant number is marked on the top left corner. (**b**) Anti-CP of TVMV and PPV immunoblot analysis of protein extracts from tissue collected from the indicated plants; MW, molecular weight; a Ponceau red-stained blot (RbcL) showing the small subunit of the Rubisco is displayed below the membrane as a loading control.

During a potyviral infection, NIapro is expressed under specific spatial–temporal conditions dictated by the virus’s needs [32]. Our results showed that two different bacterial TA systems were successfully adapted to induce a necrotic phenotype in *N. benthamiana* plants upon expression of NIa endopeptidase. Given this, we aimed to test whether the death phenotype could also be observed during a potyviral infection, laying the groundwork for the development of novel antiviral strategies. To this end, we performed agroinfiltration experiments in which we co-expressed YoeB-GFIL and YefM, or MazF-Ec, together with an infectious cDNA clone of PPV. As a negative control for the necrotic phenotype, we prepared an agroinfiltration mix in which YoeB-GFIL was substituted with an empty vector. To verify the specificity of the cleavage, we also prepared a YoeB-GFIL/YefM agroinfiltration mix in which the PPV clone was replaced with a cDNA clone for the expression of Tobacco vein mottling virus (TVMV), a closely related potyvirus that can infect *Nicotiana* plants. Eight plants were inoculated with each mix, and P19 was included in all cases to ensure the expression of the corresponding TA proteins (Figure 5).

As anticipated from previous results, the expression of the antitoxin YefM during a PPV infection did not produce any distinctive phenotype. In contrast, when the engineered YoeB-GFIL/YefM or MazF-Ec TA modules were expressed in full during a PPV infection, necrosis was observed in the agroinfiltrated area at 6dpa and became clearly apparent by 10dpa. Plants inoculated with TVMV expressing the YoeB-GFIL/YefM module exhibited a phenotype similar to the control without YoeB-GFIL, indicating that the system is species-specific and can be modulated by sequence adaptation. Western blot analysis of inoculated leaves at 10dpa confirmed the presence of TVMV and PPV in the indicated plants (Figure 5b).

## 3. Discussion

The use of bacterial elements as tools in plant biotechnology is quite widespread, ranging from the development and modification of specific plasmids for heterologous protein expression or plant transformation to the use of DNA editing enzymes for generating new traits in various plant species [33]. However, bacterial TA systems have been underutilized in plants compared to other organisms, with their application largely limited to the modulation of reproduction through cell ablation [34].

In this work, we explored two bacterial TA modules previously tested in plants, MazEF and YefM-YoeB, to investigate their biotechnological potential. We observed interesting differences suggesting distinct modes of action. MazEF appears to function as a bona fide toxin–antitoxin system in plants. Expression of MazF causes necrosis in the agroinfiltrated leaves, a phenotype that can be counteracted by the expression of the antitoxin MazE. This is in contrast to the results obtained with YefM-YoeB. In this case, expression of either component of the system alone does not cause damage to the plant cells, indicating that the toxin does not function properly in *N. benthamiana*. However, expression of the full TA complex induces a death response in the plant (Figure 1). A similar necrotic phenotype is observed when an inactive mutant toxin is expressed instead of the wild-type YoeB (Figure 2a), reinforcing the idea that the observed death response is unrelated to the action of the toxin. Given this, it is more likely that the plant itself recognizes the YefM-YoeB complex and activates an ETI-like defense response, leading to cell death. This hypothesis, which likely involves SA as part of the response, is supported by the milder necrotic phenotype detected in transgenic NahG plants compared to wild-type *N. benthamiana* expressing the YefM-YoeB TA module (Figure 2b). Additionally, SA induction by this module is corroborated by the higher levels of PR2 observed in YefM-YoeB agroinfiltrated plants compared to the controls (Figure 2c).

The fact that MazF acts as a toxin in *Nicotiana* plants, while YoeB does not, may be explained by differences in how they develop their toxicity in bacteria. MazF is an endoribonuclease that works in a ribosome-independent manner [17]. This aligns with the observation that the toxin can continue degrading mRNA in a non-bacterial context, such as the eukaryotic cell. In contrast, YoeB’s toxicity is ribosome-dependent [20], which suggests that the shift from bacteria to plants may not support its activity as a blocker of translation initiation. These mechanistic differences, however, do not account for the contrasting results reported in *A. thaliana* with the YefM-YoeB module [18,19]. Given that YoeB’s action is ribosome-dependent, it is possible that different hosts present different compatibilities that could render the toxin either active or inactive. It is also possible that the green fluorescence protein (GFP) tag on the toxin used in the *Arabidopsis* study may affect its interaction with the plant, potentially influencing mRNA translation blockage or eliciting an ETI-like response that is not triggered by wild-type YoeB. The significance of interactions between the plant and the module components is further underscored by the unexplained enhanced growth phenotype observed in *Arabidopsis* plants transformed with the antitoxin YefM gene [19].

The different behavior of similar bacterial TA systems adds complexity to the situation but also presents an opportunity to develop diverse biotechnological tools that might be suited to various contexts. In this work, we successfully adapted the MazEF system used in animal cells for plants, incorporating the cleavage site of a plant viral endopeptidase and modifying the ER membrane anchor, as reported in [29] (Figure 3). For YoeB, we explored the use of aggregation tags to render the protein inactive but still functional. While such tags have been extensively investigated in bacterial systems [31,35,36], they have not been tested in plants before. The positive results obtained (Figure 4) validate the modified YefM-YoeB system and highlight the potential of using aggregation tags as an alternative approach for expressing harmful proteins in plants or for other biotechnological applications, such as plant protein purification [37].

Antiviral defenses that involve a death phenotype are naturally exploited by plants through a hypersensitive response (HR), which is controlled by resistance genes [38]. Biotechnology has also developed antiviral tools based on modifying existing HR pathways, linking them to protease activation [39,40]. In our work, we modified two TA systems to place them under the control of the endopeptidase NIapro from PPV, demonstrating that they are activated not only upon transient expression of the protease but also during a PPV infection (Figure 5). These results highlight the great potential of these engineered modules as antiviral tools, offering two significant benefits: (i) they can likely be used in various contexts, particularly the MazF-Ec system, due to their ability to work independently of other factors, and (ii) they can be easily adapted to target specific viruses by simple modification of the NIapro cleavage site. Combining these tools with new commercial biofertilizers [41] and modern pest management techniques related to control strategies [42] and artificial intelligence [43] could significantly enhance protection against viral pathogens. However, it is important to acknowledge that these systems are not yet fully optimized for viral infection. Under the specific conditions tested, the virus was not stopped at the agroinfiltrated site and was able to spread systemically through the plant. Thus, while these systems serve as a proof-of-concept, further optimization is needed to develop effective antiviral systems. Generating transgenic lines that constitutively express the modified TA modules and testing infection conditions that more closely mimic natural scenarios, such as aphid transmission, will likely be necessary to fully assess the potential of these systems as plant antiviral control strategies.

## 4. Materials and Methods

### 4.1. Plasmids

*MazE, MazF, YoeB, YefM, MazF-EC, YoeB-GFIL*: Sequences for these plasmids (File S1 in the Supplementary Materials) were synthesized as DNA strings (Invitrogen, Waltham, MA, USA) with gateway tails for cloning by BP recombination into pDONR207 plasmid (Invitrogen). LR recombination of these plasmids followed, using as destination vectors pGWB702Ω and pGWB402Ω for YefM-YoeB and MazEF components, respectively [44].

NIa, NIa*: Plasmid pGWB718-NIa carrying the sequence for the PPV protease was reported [45]. The NIa mutant was synthesized by overlapping the PCR with primer pairs 1F-2R and 3F-4R (File S1 in the Supplementary Materials) to introduce the mutation in the catalytic site. A second PCR using the first PCR products as templates and using primer pair 1F-4R was performed before cloning this product by Gateway BP recombination into the pDONR207 plasmid (Invitrogen). LR recombination followed, using pGWB718 as a destination vector [44]. PCR amplification was performed with Phusion (Thermo Fisher Scientific, Waltham, MA, USA) following the manufacturer´s instructions.

*PPV, TVMV, P19*: Viral vectors used for PPV (PPVr) and TVMV inoculations have been reported [46,47], and plasmid pBIN61:p19 for expression of tombusviral RNA silencing suppressor P19 was kindly provided by Prof. David Baulcombe (University of Cambridge, Cambridge, UK).

### 4.2. Agroinfiltration and Images

*N. benthamiana* plants were grown in a greenhouse with a 16 h light/8 h dark photoperiod kept at a temperature range of 19–23 °C. Plants with 4–5 leaves were infiltrated as described [48] with *A. tumefaciens* strain C58C1-313 [49] carrying the indicated plasmids and using an OD_600_ of 0.4 for each construct. Photos were taken with a digital camera, Nikon D3X, at the indicated times.

### 4.3. Western Blot Analysis

Plant tissue grounded in liquid nitrogen was used to prepare protein extracts with extraction buffer (125 mM Tris-HCl, 2% SDS, 6 M urea, 5% β-mercaptoethanol, 10% glycerol, 0.05% bromophenol blue, pH 7.5) using 2 mL/g of tissue. Proteins were resolved by electrophoresis on 12% acrylamide sodium dodecyl sulfate polyacrylamide gels and electroblotted onto nitrocellulose membranes. Anti-PR2, anti-CP of PPV, and anti-CP of TVMV serum were used as primary antibodies at 1:10,000, 1:100,000, and 1:10,000 dilutions, respectively. Horseradish peroxidase-conjugated goat anti-rabbit IgG (Merck, Darmstadt, Germany) was used as a secondary antibody for protein detection (1:10,000 dilution). Immunostained proteins were visualized by enhanced chemiluminescence detection with Clarity Western ECL Substrate (Bio-Rad, Hercules, CA, USA). Bands were quantified with Fiji program (v2.15.1) [50], and an analysis of variance (n = 4, *p* < 0.05) (one-way ANOVA) followed by Tukey´s test was performed using IBM SPSS Statistics (v29.0.2).

## 5. Conclusions

Bacterial TA systems have been underutilized in plants despite their demonstrated potential in other organisms. This study explores various constructs and engineers molecular switches based on TA modules that can be activated by viral endopeptidases. These constructs pave the way for the development of diverse applications, including novel antiviral strategies. Although further optimization is necessary to fully realize their antiviral potential, they lay the groundwork for future research in the field.

## Data Availability

Data are contained within the article.

## Supplementary Materials

The following supporting information can be downloaded at: https://www.mdpi.com/article/10.3390/ijms251910449/s1.
