# Supplementary material for: Use of Bacterial Toxin–Antitoxin Systems as Biotechnological Tools in Plants
Source: Int J Mol Sci. 2024 Sep 27;25(19):10449. doi: 10.3390/ijms251910449 (PMC11476816; doi:10.3390/ijms251910449)
Supplement: Supplementary file 1 [file ijms-25-10449-s001.zip › ijms-3196064-supplementary.pdf]

File S1

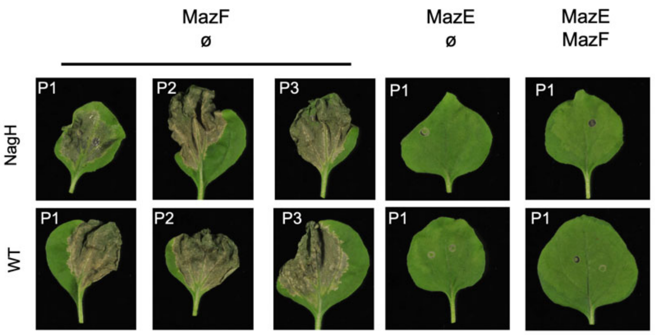

Figure S1. MazEF system in NahG and wild type *Nicotiana benthamiana* plants. Photos from the plants agroinfiltrated with MazF, MazE or MazF and MAzE were taken at 8dpa. Three representative images from eight plants are shown in the case of MazF. All mixes were prepared including P19 as RNA silencing suppressor. Plant number is marked on the top left corner.

Synthesized constructs

MazE

GGGGACAAGTTTGTACAAAAAAGCAGGCTTCATGATCCACAGTAGCGTAAAGCGTTGGGGAAA
TTCACCGGCGGTGCGGATCCCGGCTACGTTAATGCAGGCGCTCAATCTGAATATTGATGATGAAG
TGAAGATTGACCTGGTGGATGGCAAATTAATTATTGAGCCAGTGCGTAAAGAGCCCGTATTTACG
CTTGCTGAACTGGTCAACGACATCACGCCGGAAAACCTCCACGAGAATATCGACTGGGGAGAG
CCGAAAGATAAGGAAGTCTGGTAACCCAGCTTTCTTGTACAAAGTGGTCCCC

MazF

GGGGACAAGTTTGTACAAAAAAGCAGGCTTCATGGTAAGCCGATACGTACCCGATATGGGCGAT
CTGATTTGGGTTGATTTTGACCCGACAAAGGGTAAGTATGCACTTAAAGAGTATGTGTGGAAAA
AGTTCTTCATAACCACTTCTAGTAGAAAAAAATAACAAGGAAACATGAAGCTATTTCCTACTAGC
TTGTCCAAAATCTTATTTTTAACTCAAACTAATTTTAACTTGTGGAAGAAAACAACTTCTTTACATT
TTATAATCTGAGCATTGGTGTGGGGTCCTTAGGTTCAATCTTTGAAATTGTGCAGGGTAGCGAGC
AAGCTGGACATCGTCCAGCTGTTGTCCTGAGTCCTTTCATGTACAACAACAAAACAGGTATGTGT
CTGTGTGTTCCTTGTACAACGCAATCAAAAGGATATCCGTTCGAAGTTGTTTTATCCGGTCAGGA
ACGTGATGGCGTAGCGTTAGCTGATCAGGTAAAAAGTATCGCCTGGCGGGCAAGAGGAGCAAC
GAAGAAAGGAACAGTTGCCCCAGAGGAATTACAACTCATTAAAGCCAAAATTAACGTACTGATT
GGGTAGCCCAGCTTTCTTGTACAAAGTGGTCCCC

MazF-Ec

GGGGACAAGTTTGTACAAAAAAGCAGGCTTCATGGTAAGCCGATACGTACCCGATATGGGCGAT
CTGATTTGGGTTGATTTTGACCCGACAAAGGTAAGTATGCACTTAAAGAGTATGTGTGGAAAAA
GTTCTTCATAACCACTTCTAGTAGAAAAAAATAACAAGGAAACATGAAGCTATTTCCTACTAGCTT
GTCCAAAATCTTATTTTTAACTCAAACTAATTTTAACTTGTGGAAGAAAACAACTTCTTTACATTTT
ATAATCTGAGCATTGGTGTGGGGTCCTTAGGTTCAATCTTTGAAATTGTGCAGGGTAGCGAGCA
AGCTGGACATCGTCCAGCTGTTGTCCTGAGTCCTTTCATGTACAACAACAAAACAGGTATGTGTC
TGTGTGTTCCTTGTACAACGCAATCAAAAGGATATCCGTTCGAAGTTGTTTTATCCGGTCAGGAA
CGTGATGGCGTAGCGTTAGCTGATCAGGTAAAAAGTATCGCCTGGCGGGCAAGAGGAGCAACG

AAGAAAGGAACAGTTGCCCCAGAGGAATTACAACTCATTAAAGCCAAAATTAACGTACTGATTG
GGGGAGGATCAAACGTTGTTGTGCACCAAGCTGACGAAGGAGGCGGAGGCTCACGTAAAGAG
CCCGTATTTACGCTTGCTGAACTGGTCAACGACATCACGCCGGAAAACCTCCACGAGAATATCG
ACTGGGGAGAGCCGAAAGATAAGGAAGTCTGGTCATCTGGAGGATCAGGAGGAGGTTCAGGT
TCAGGAGGAGACAAGACCTCTGAATTCATAATCAAGCTCCTCCAGTTCCTCGTACCCCTTGCCAT
TCTTGGTTTAGCAGTCGGAATCCGTATCTACACCAAATCAGGGTAGCCCAGCTTTCTTGTACAAA
GTGGTCCCC

YefM
GGGGACAAGTTTGTACAAAAAAGCAGGCTTCATGGAAGCTGTGCTGTACTCCACTTTTCGGAA
CCACCTGAAGGACTACATGAAGAAGGTGAACGACGAGTTCGAGCCTCTTACCGTGGTGAACAA
GAACCCTGATGAGGATATCGTGGTGCTGAGCAAGTCTGAGTGGGACTCTATTCAAGAGACTCTG
CGGATCGCCCAGAACAAAGAGCTGTCTGATAAGGTTCTGCGTGGCATGGCTCAAGTGAGAGCT
GGTTCTACTCAGGTTCACGTGATCGAAGAGTAGCCCAGCTTTCTTGTACAAAGTGGTCCCC

YoeB
GGGGACAAGTTTGTACAAAAAAGCAGGCTTCATGCTGCTGAAGTTCACCGAAGATGCTTGGGC
TGATTACTGCTACTGGCAGAACCAGGACAAGAAAACCCTGAAGCGGATCAACAAGCTGATCAA
GGTAAGTATGCACTTAAAGAGTATGTGTGGAAAAAGTTCTTCATAACCACTTCTAGTAGAAAAAA
ATAACAAGGAAACATGAAGCTATTTCCTACTAGCTTGTCCAAAATCTTATTTTTAACTCAAACTAAT
TTTAACTTGTGGAAGAAAACAACTTCTTTACATTTTATAATCTGAGCATTGGTGTGGGGTCCTTAG
GTTCAATCTTTGAAATTGTGCAGGATATCCAGAGGGACCCTTTCACCGGTATTGGTAAGCCTGAG
CCTCTGAAGTACGATTACCAAGGCGCTTGGTCTAGAAGGATCGATGCTGAGAACCGGCTGATCT
ACATGATGGATGGTGACTCTGTGGCCTTCCTGAGCTTCAAGGATCACTACCCCAGCTTTCTTGTA
CAAAGTGGTCCCC

YoeB-GFIL
GGGGACAAGTTTGTACAAAAAAGCAGGCTTCATGCTGCTGAAGTTCACCGAAGATGCTTGGGC
TGATTACTGCTACTGGCAGAACCAGGACAAGAAAACCCTGAAGCGGATCAACAAGCTGATCAA
GGGTAAGTATGCACTTAAAGAGTATGTGTGGAAAAAGTTCTTCATAACCACTTCTAGTAGAAAAA
AATAACAAGGAAACATGAAGCTATTTCCTACTAGCTTGTCCAAAATCTTATTTTTAACTCAAACTA
ATTTTAACTTGTGGAAGAAAACAACTTCTTTACATTTTATAATCTGAGCATTGGTGTGGGGTCCTT
AGGTTCAATCTTTGAAATTGTGCAGGATATCCAGAGGGACCCTTTCACCGGTATTGGTAAGCCTG
AGCCTCTGAAGTACGATTACCAAGGCGCTTGGTCTAGAAGGATCGATGCTGAGAACCGGCTGAT
CTACATGATGGATGGTGACTCTGTGGCCTTCCTGAGCTTCAAGGATCACTACAACGTTGTTGTGC
ACCAAGCTGACGAACCAACCCCACCGACGACTCCTACTCCGCCCACAACTCCGACCCCAACTCC
GGGTTTCATTCTGGGTTTCATTCTGTAGCCCAGCTTTCTTGTACAAAGTGGTCCCC

Primers
1F: 5´- ggggacaagtttgtacaaaaaagcaggctccatggcaGGCTTCAATCGTAGGCAAAGAC- 3´
2R: 5´- GGGTAATCCGgcATGACCATCC - 3´
3F: 5´- GGATGGTCATgcCGGATTACCC - 3´
4R: 5´- ggggaccactttgtacaagaaagctgggttaCTGAGTGTAAACAAATTCCCC- 3´
